# Association between general anesthesia and contrast-induced encephalopathy after endovascular treatment on neurovascular diseases

**DOI:** 10.3389/fneur.2023.1146194

**Published:** 2023-05-12

**Authors:** Zhihong Zhong, Hongyang Ni, Jun Zhu, Hong Jiang, Jinqing Hu, Dong Lin, Liuguan Bian

**Affiliations:** Department of Neurosurgery, Ruijin Hospital, Shanghai Jiao Tong University School of Medicine, Shanghai, China

**Keywords:** contrast-induced encephalopathy, anesthesia, endovascular treatment, risk factor, propofol

## Abstract

**Objective:**

Contrast-induced encephalopathy (CIE) is a rare neurological complication that can occur in the context of various endovascular procedures. Although many potential risk factors for CIE have been reported, it is still unclear whether anesthesia is a risk factor for the occurrence of CIE. The goal of this study was to investigate the incidence of CIE in patients who underwent endovascular treatment under different anesthesia methods and anesthetics administration and to explore whether general anesthesia was a potential risk factor for CIE.

**Methods:**

We retrospectively reviewed available clinical data from 1,043 patients with neurovascular diseases undergoing endovascular treatment between June 2018 and June 2021 in our hospital. A propensity score-based matching strategy and logistic regression were used to analyze the association between anesthesia and the occurrence of CIE.

**Results:**

In this study, we implemented the embolization of intracranial aneurysm in 412 patients, stent implantation of extracranial artery stenosis in 346, stent implantation of intracranial artery stenosis in 187, embolization of cerebral arteriovenous malformation or dural arteriovenous fistula in 54, endovascular thrombectomy in 20, and other endovascular treatments in 24. A total of 370 patients (35.5%) received treatment under local anesthesia, while the remaining 673 (64.5%) underwent treatment under general anesthesia. In total, 14 patients were identified as CIE, resulting in a total incidence rate of 1.34%. After propensity score-based matching of anesthesia methods, the occurrence of CIE was significantly different between the general anesthesia and local anesthesia group (*P* = 0.007). After propensity score-based matching of CIE, the anesthesia methods were significantly different between the two groups. Pearson contingency coefficients and logistic regression showed a significant correlation between general anesthesia and the risk of CIE.

**Conclusion:**

General anesthesia might be a risk factor for CIE, and propofol might be associated with the increased occurrence of CIE.

## Introduction

Contrast-induced encephalopathy (CIE) is a rare neurological complication following the administration of contrast agents in various angiographic procedures. It has been reported to occur in 1.7–3.6% of neurological endovascular procedures, including cerebral angiography, coil embolization, and endovascular thrombectomy procedure (1–3). The clinical manifestations mainly include encephalopathy, cortical blindness, motor deficit, decreased vigilance, aphasia, headache, and epileptic seizures (4).

The mechanisms of CIE remain unclear, and it is speculated that increased permeability of the blood–brain barrier (BBB), hyperosmolarity, and direct neurotoxicity from the contrast agents may lead to CIE (5). The potential risk factors for CIE may include chronic hypertension, renal dysfunction, history of stroke, high-contrast osmolality, and overdose of contrast medium (3–8). However, it is still unclear whether anesthesia is a risk factor for the occurrence of CIE, considering that anesthetics (e.g., propofol) may affect the permeability of BBB or have direct neurotoxicity. Therefore, our study aimed to investigate the incidence of CIE in patients who underwent endovascular treatment under different anesthesia methods and anesthetics administration and to explore whether general anesthesia was the potential risk factor for CIE.

## Methods

### Data collection

The clinical data available in our database collected from patients with neurovascular diseases undergoing endovascular treatment were reviewed from June 2018 to June 2021. The participants were fully informed, and signed consent forms were obtained. The study was approved by the medical ethics committee of Ruijin Hospital, affiliated with Shanghai Jiao Tong University School of Medicine.

Clinical data, including age, sex, symptoms, neuroimaging, anesthesia methods, treatment, operation duration, past medical history, and outcomes of patients, were collected and analyzed. The operation duration was defined as the time period from the start of anesthesia to the end of the operation.

### Anesthesia and contrast medium

The patients were arranged to receive local anesthesia or general anesthesia, depending on the type and location of the lesion and also by the request of the patient. Generally, the patients with intracranial aneurysm, cerebral arteriovenous malformation, intracranial artery stenosis, dural arteriovenous fistula, or acute ischemic stroke were recommended to undergo general anesthesia. Otherwise, the patients with extracranial artery stenosis (i.e., stenosis of the initial segment of the internal carotid artery) or extracranial arterial dissection, or the patients who would undergo embolization of feeding arteries of convex meningioma, were recommended to receive local anesthesia. In patients with local anesthesia, only 2% lidocaine was injected into the inguinal region locally, while in patients with general anesthesia, anesthetic drugs were given intravenously by experienced anesthetists. Generally, the anesthetic drugs included sedatives (propofol and dexmedetomidine), muscular relaxants (rocuronium and cisatracurium), and analgesics (fentanyl, sufentanil, and remifentanil). The choice of these abovementioned anesthetic drugs depended on the need for anesthesia. The dose of each drug administrated was based on the body weight of the patients. Additionally, if the patients stayed in prolonged intubation after endovascular treatment, dexmedetomidine would be used for sedation, and the dosage of dexmedetomidine would also be documented (Supplementary Table 1).

A non-ionic contrast medium was administrated intravenously in all cases during endovascular treatment. The main contrast medium used was iopamidol or iodixanol. In addition, the contrast dose of a CT angiography (CTA), in which a non-ionic contrast medium was used, would also be included in the total contrast dose if the CTA was performed within 24 h prior to endovascular treatment.

### Diagnosis of CIE

**Figure 1:**
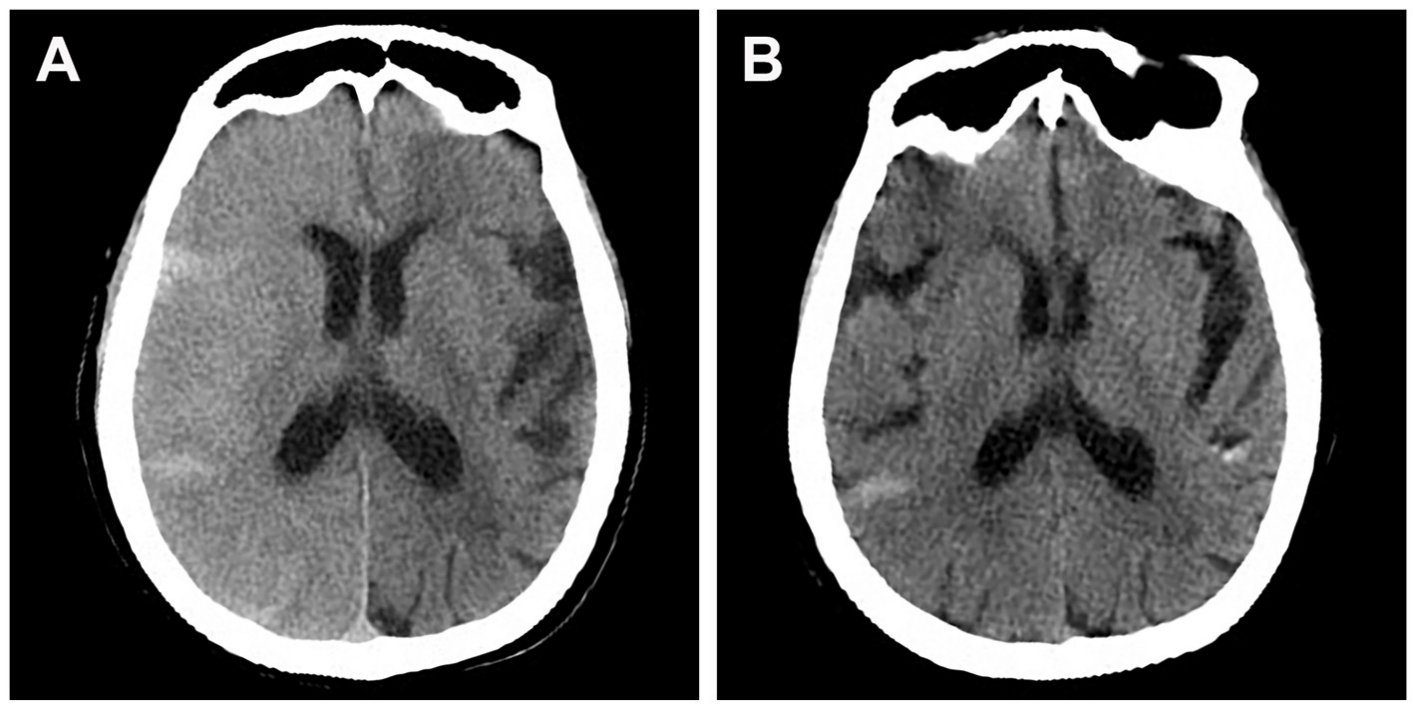
Brain CT scan of a patient who presented with lethargy, aphasia, and contralateral side limb weakness after stent-assisted embolization of posterior communicating aneurysm. **(A)** CT scan performed 2 h after symptom onset showing diffuse right cerebral edema and contrast staining. **(B)** CT scan performed after clinical resolution (36 h after symptom onset) showing resolution of cerebral edema.

**Figure 2:**
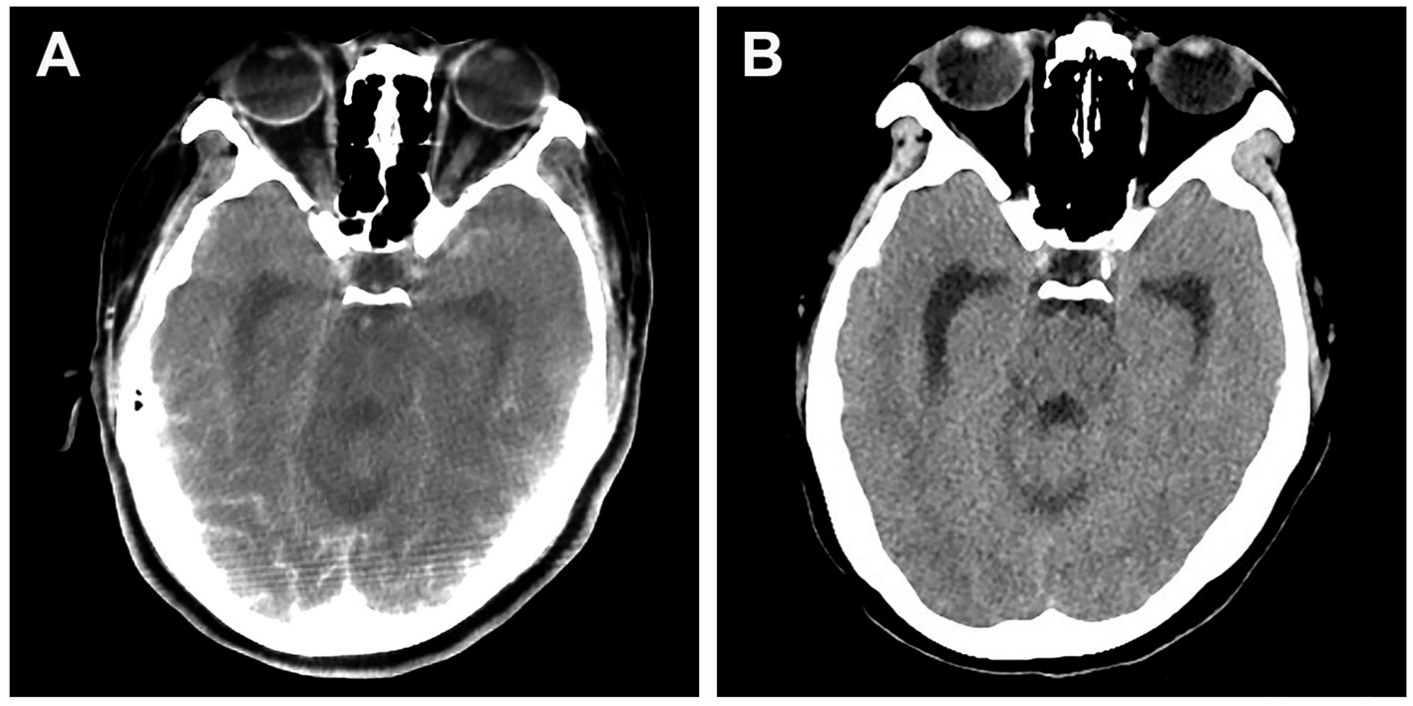
Brain CT scan of a patient who presented with cortical blindness after stent-assisted embolization of vertebral aneurysm. **(A)** CT scan performed 2 h after symptom onset showing significant contrast staining at the occipital lobes. **(B)** CT scan performed after clinical resolution (24 h after symptom onset) showing effacement of contrast staining.

The patient was diagnosed with CIE if both the clinical and radiological criteria were fulfilled within 24 h following endovascular treatment. The clinical criteria were unequivocal clinical deterioration (i.e., a decrease of ≥2 in the Glasgow Coma Scale score and a decrease in muscle strength) and/or onset of new neurological symptoms (i.e., disorientation and cortical blindness) that could not be explained by recurrent ischemic stroke, hemorrhage, and metabolic abnormalities. The radiological criterion was regional cerebral edema accompanied by contrast staining, which was defined as the presence of a hyperdense lesion in the brain parenchyma or subarachnoid space that persisted on follow-up neuroimaging (3). The patients receiving general anesthesia were neurologically evaluated immediately after awakening from anesthesia and at the onset of the new event. Neuroimaging was arranged in all of them at an appropriate time for ruling out stroke or hemorrhage (Figures 1, 2).

### Statistical analysis

A propensity score-based matching strategy was used to select a control group. For patients who underwent treatment with general anesthesia, controls with local anesthesia were matched by age, sex, hypertension, diabetes, renal dysfunction, history of stroke, and dose of contrast agent to the corresponding case with a ratio of 1:1 with the nearest propensity score (caliper width of 0.10). The differences between general and local anesthesia after matching were shown by standardized mean difference (SMD). All continuous data were presented as mean ± standard deviation (SD) and compared by *t*-test. All categorical data were presented as N (%) and compared by chi-square. The correlation between the anesthesia methods and the event of CIE was analyzed by Pearson's contingency coefficients.

Considering the low prevalence of CIE in the real world and the limited events of CIE in our data, the controls were matched by age, sex, hypertension, diabetes, renal dysfunction, history of stroke, and dose of contrast agent to the patients with CIE to further study the association between the anesthesia methods and risk of CIE. Each patient with CIE was matched to four control subjects with the nearest propensity score (caliper width of 0.10). All continuous data were presented as mean ± standard deviation (SD) and compared by *t*-test. All categorical data were presented as N (%) and compared by chi-square. Logistic regression was used to estimate the odds ratio (OR) of CIE and its 95% confidence interval (95% CI). The additional sensitivity analysis comprised the risk of CIE associated with propofol dosage in unit weight. We calculated the median propofol dosage in unit weight in patients with general anesthesia first. The patients were grouped into three groups: never used, used less than median propofol dosage in unit weight, and used more than median propofol dosage in unit weight. The test for trend was based on variables containing a median value for each quintile.

A *p*-value of < 0.05 (two-tailed) was considered statistically significant. R (version 4.1.1) and R Studio (version 1.1.442) were used to perform the statistical analysis.

## Results

### All patients' characteristics

During the study period, 1,043 patients underwent endovascular treatment, among whom 14 patients were diagnosed with CIE, resulting in a total incidence rate of 1.34% (Table 1). Before endovascular treatment, a CT angiography was given to 453 patients, among whom 26 patients had the CT angiography within 24 h prior to endovascular treatment. In these 1,043 patients, we implemented embolization of intracranial aneurysm in 412, stent implantation of extracranial artery stenosis in 346, stent implantation of intracranial artery stenosis in 187, embolization of cerebral arteriovenous malformation or dural arteriovenous fistula in 54, endovascular thrombectomy in 20, and other endovascular treatments in 24. Out of the 1,043 patients, 370 received treatment under local anesthesia, while the remaining 673 patients underwent treatment under general anesthesia. In total, 18 patients stayed in prolonged intubation after endovascular treatment under general anesthesia and received an intravenous injection of dexmedetomidine by micropump for sedation. The duration of sedation in these 18 patients varied from 8 h to 36 h.

**Table 1:** Characteristics of patients with CIE.

**Case**	**Age**	**Sex**	**Medical history**	**Diagnosis**	**Procedure**	**Encephalopathy signs**	**Clinical resolution**
1	71	F	HTN	PICA An	SAC	Cortical blindness	POD 2
2	63	M	HTN	PICA An, ICA St	SAC, stenting	Agitation, CS limb weakness	POD 2
3	57	F	HTN, DM	ICA An	SAC	Aphasia, CS limb weakness	POD 2
4	68	M	/	ICA St	Stenting	Aphasia, CS limb weakness, seizure	POD 4
5	76	M	Stroke	MCA St	Stenting	Lethargy, CS limb weakness	POD 3
6	64	F	HTN	PComA An	SAC	Lethargy, aphasia, CS limb weakness	POD 2
7	57	M	HTN, stroke	BA An	SAC	Cortical blindness	POD 3
8	50	F	/	ICA An	SAC	CS limb weakness, seizure	POD 6
9	70	M	AF, stroke	VA An	SAC	Cortical blindness	POD 1
10	44	F	/	ICA An	SAC	Aphasia, CS limb weakness	POD 2
11	73	M	/	VA St	Stenting	Cortical blindness	POD 3
12	51	M	HTN	VA An	SAC	Cortical blindness	POD 1
13	66	M	HTN, DM, stroke	VA An	SAC	Disorientation, cortical blindness	POD 3
14	74	M	HTN, DM, CAD	ICA St	Stenting	Agitation, CS limb weakness	POD 3
**Case**		**Contrast dose (mL)**	**Operation duration (h)**	**Anesthesia**	**Sedative**	**Muscular relaxant**	**Analgesic**
1		110	2.50	GA	Pro 650 mg, Dex 15 ug	Cis 32 mg	Suf 15 ug
2		220	2.50	GA	Pro 870 mg, Dex 20 ug	Cis 32 mg	Fen 0.2 mg
3		95	2.50	GA	Pro 650 mg	Cis 35 mg	Fen 0.2 mg
4		85	1.50	GA	Pro 590 mg	Cis 20 mg	Fen 0.1 mg, Rem 0.6 mg
5		90	2.50	GA	Pro 650 mg, Dex 30 ug	Roc 50 mg	Fen 0.2 mg, Rem 1.5 mg
6		155	2.00	GA	Pro 650 mg, Dex 25ug	Roc 50 mg	Fen 0.2 mg, Rem 1.6 mg
7		115	3.00	GA	Pro 1,500 mg	Cis 3 2 mg	Fen 0.2 mg, Rem 1.4 mg
8		100	1.75	GA	Pro 450 mg	Cis 10 mg	Fen 0.1 mg, Rem 0.8 mg
9		140	3.25	GA	Pro 1250 mg	Roc 40 mg	Fen 0.15 mg, Rem 1.5 mg
10		90	1.50	GA	Pro 450 mg	Cis 12 mg	Fen 0.3 mg
11		95	2.00	GA	Pro 650 mg	Roc 37.5 mg	Fen 0.15 mg, Rem 1.1 mg
12		90	2.00	GA	Pro 700 mg	Roc 60 mg	Fen 0.1 mg, Rem 0.9 mg
13		90	2.75	GA	Pro 850 mg	Roc 80 mg	Fen 0.25 mg, Rem 1.0 mg
14		85	0.90	LA	/	/	/

AF, atrial fibrillation; An, aneurysm; BA, basilar artery; CAD, coronary artery disease; CIE, contrast-induced encephalopathy; Cis, cisatracurium; CS, contralateral side; Dex, dexmedetomidine; DM, diabetes mellitus; Fen, fentanyl; GA, general anesthesia; HTN, hypertension; ICA, internal carotid artery; LA, local anesthesia; MCA, middle cerebral artery; PComA, posterior communicating artery; PICA, posterior inferior cerebellar artery; POD, postoperative day; Pro, propofol; Rem, remifentanil; Roc, rocuronium; SAC, stent-assisted coiling; St, stenosis; Suf, sufentanil; VA, vertebral artery.

The incidence of CIE was 0.27% (1/370) and 1.93% (13/673) in the local anesthesia and general anesthesia groups, respectively. Also, a higher dose of the contrast medium was used in the general anesthesia group than in the local anesthesia group. The baseline characteristics of the two groups of patients are presented in Table 2.

**Table 2:** Baseline characteristics of the 1,043 patients.

**Characteristics**	**Total**	**GA**	**LA**
Age, X ± SD, y	61.9 ± 11.8	58.8 ± 12.4	67.4 ± 8.4
Sex, male, *N* (%)	619 (59.3)	312 (46.4)	307 (83.0)
History of hypertension, *N* (%)	720 (69.0)	434 (64.5)	286 (77.3)
History of diabetes mellitus, *N* (%)	329 (31.5)	187 (27.8)	142 (38.4)
History of heart disease, N (%)	160 (15.3)	70 (10.4)	90 (24.3)
History of renal dysfunction, *N* (%)	51 (4.9)	26 (3.9)	25 (6.8)
History of stroke, *N* (%)	455 (43.6)	298 (44.3)	157 (42.4)
Contrast dose, X ± SD, mL	107.0 ± 32.2	111.4 ± 32.7	99.1 ± 29.6
Operation duration, X ± SD, h	1.8 ± 0.9	2.3 ± 0.8	1.0 ± 0.3
Incidence of CIE, *N* (%)	14 (1.3)	13 (1.9)	1 (0.3)

CIE, contrast-induced encephalopathy; GA, general anesthesia; LA, local anesthesia; SD, standard deviation.

### Propensity score-based matching of anesthesia methods

After propensity score-based matching of anesthesia methods, 290 patients with general anesthesia were matched to 290 patients with local anesthesia. The variables of age, sex, hypertension, diabetes mellitus, nephropathy, history of stroke, and dose of contrast medium were more balanced between the two groups after matching (Table 3). The occurrence of CIE was significantly different between the general anesthesia and local anesthesia group (*P* = 0.007, Table 3). Pearson's contingency coefficients showed a significant correlation between general anesthesia and the event of CIE (*r* = 0.117, *P* = 0.004).

**Table 3:** Characteristics of general anesthesia and their matched controls.

**Characteristics**	**GA (*N* = 290)**	**LA (*N* = 290)**	**P-value**	**SMD**
Age, X ± SD, y	64.0 ± 10.5	65.1 ± 7.7	0.118	0.096
Sex, male, *N* (%)	213 (73.4)	227 (78.3)	0.174	0.097
Hypertension, *N* (%)	211 (72.8)	217 (74.8)	0.571	0.043
Diabetes mellitus, *N* (%)	105 (36.2)	108 (37.2)	0.796	0.023
History of stroke, *N* (%)	127 (43.8)	133 (45.9)	0.616	0.042
Renal dysfunction, *N* (%)	13 (4.5)	19 (6.6)	0.275	0.107
Contrast dose, X ± SD, mL	105.5 ± 28.4	103.5 ± 31.5	0.443	0.074
Incidence of CIE, N (%)	8 (2.7)	0 (0.0)	0.007	/

CIE, contrast-induced encephalopathy; GA, general anesthesia; LA, local anesthesia; SD, standard deviation; SMD, standardized mean difference.

### Propensity score-based matching of CIE

To further study the association between the anesthesia methods and the risk of CIE, a propensity score-based matching of CIE was performed, and 14 patients with CIE were matched to 54 patients without CIE. The variables of age, sex, hypertension, diabetes mellitus, nephropathy, history of stroke, and dose of contrast agent were more balanced between the two groups after matching (Table 4). The anesthesia methods were significantly different between the two groups (*P* = 0.027, Table 4).

**Table 4:** Characteristics of CIE and their matched controls.

**Characteristics**	**CIE (*N* = 14)**	**Control (*N* = 54)**	**P-value**	**SMD**
Age, X ± SD, y	63.4 ± 9.9	63.8 ± 10.4	0.842	0.090
Sex, male, *N* (%)	9 (64.3)	39 (72.2)	0.743	0.186
Hypertension, *N* (%)	8 (57.1)	35 (64.8)	0.596	0.108
Diabetes mellitus, *N* (%)	3 (21.4)	10 (18.5)	1.000	0.087
History of stroke, *N* (%)	4 (28.6)	17 (31.5)	1.000	0.040
Renal dysfunction, *N* (%)	0 (0.0)	0 (0.0)	/	0.000
Contrast dose, X ± SD, mL	111.4 ± 37.6	107.5 ± 30.0	0.680	0.083
GA, *N* (%)	13 (92.9)	33 (61.1)	0.027	/
Propofol dose, X ± SD, mL	707.9 ± 94.7	497.3 ± 68.6	0.083	/

CIE, contrast-induced encephalopathy; GA, general anesthesia; SD, standard deviation; SMD, standardized mean difference.

Further logistic analysis showed that the risk of CIE after general anesthesia was 7.273 times higher than that after local anesthesia (OR = 8.273, *P* = 0.049). Moreover, we analyzed the association between propofol dosage in unit weight and CIE and demonstrated a dose–response manner. An increased risk was seen for the lower dosage ( ≤ 11.1 mg/kg) in trend (OR = 7.412, *P* = 0.076) than the higher dosage (>11.1 mg/kg) (OR = 9.188, *P* = 0.048), indicating that the risk of CIE increased in a statistically significant dose-dependent pattern with increased propofol dosage in unit weight (P trend = 0.035).

## Discussion

In the present study, the overall incidence of CIE in the 1,043 patients who underwent endovascular treatment was 1.34%, which was similar to the incidence reported in previous literature (1–3). The incidences of CIE were 0.27% and 1.93% in the local anesthesia and general anesthesia groups, respectively, and the latter incidence was significantly higher than the former. After propensity score-based matching of anesthesia methods, the occurrence of CIE was still significantly different between the general anesthesia and local anesthesia groups (*P* = 0.007). This difference in incidence between these two groups indicated that anesthesia methods and/or anesthetics might be associated with the occurrence of CIE.

In order to further study the association between anesthesia methods and the risk of CIE, we performed a propensity score-based matching of CIE. Similarly, the anesthesia methods were significantly different between the CIE and control groups after balancing the variables of risk factors. Further logistic regression analysis also showed a positive association between general anesthesia and the risk of CIE, indicating that general anesthesia might be a risk factor for CIE. To the best of our knowledge, this was the first comprehensive analysis of the relationship between anesthesia and CIE in patients with neurovascular diseases undergoing endovascular treatment. The propensity score-based matching technique performed in this study for patient cohort selection has provided assurance for the better control of confounders and therefore may add more strength to the validation of our results.

Increased permeability of the BBB, hyperosmolarity, and direct neurotoxicity from the contrast agents are considered to be involved in the occurrence of CIE (5). During the general anesthesia procedure in our study, the sedatives, muscular relaxants, and analgesics were intravenously administrated to the patients, and propofol was used in all these patients. The lipophilic property of propofol allows it to cross the BBB rapidly and distribute into the central nervous system (9). Remsen et al. reported that propofol was observed to provide better disruption of the BBB in tumor-bearing rats than isoflurane did, and thus, drug delivery to tumors and the brain tissue around the tumors was significantly improved with propofol anesthesia (10). Fortin et al. also observed that the neurotoxicity of chemotherapy delivery increased significantly in BBB-disruption rats with propofol anesthesia (11). Thus, we further explored whether the dosage of propofol in unit weight was associated with CIE in our studies and demonstrated a dose-dependent pattern with increased propofol dosage in unit weight. Combining these findings with our observation, we hypothesized that propofol might be associated with the increased occurrence of CIE in the general anesthesia group, although the administration of propofol was in the normal dose range in our study. The possible reason might involve the transiently increased permeability or disruption of BBB caused by propofol, which increased the amount of contrast distributed in the brain tissue. However, the sample in our study was small, especially in the case of CIE, and thus, results from studies with larger samples are needed to support our findings.

Fentanyl was another common anesthetic agent used in most of our patients with general anesthesia. However, there is little evidence of changes in the permeability of the *in vitro* BBB caused by fentanyl (12). Similarly, rare evidence showed that other sedatives, muscular relaxants, and analgesics were related to the disruption or increased permeability of BBB. However, whether these anesthetic drugs will contribute to the occurrence of CIE needs to be investigated in further studies with larger sample sizes.

There is still no guideline recommendation for the treatment of CIE. Considering the potential risk factors for CIE, including chronic hypertension, renal dysfunction, history of stroke, high-contrast osmolality, overdose of contrast medium, and anesthetics, the patients at a high risk of CIE should receive a minimized dose of contrast and sedatives (13). CIE usually alleviates after several days to weeks of supportive management (3). The therapy of vigorous hydration, steroids, and mannitol is controversial. They have been reported to be used to treat severe CIE (14–17). However, Quintas-Neves et al. found no relation between the institution of treatments and complete clinical recovery in a systematic review (4).

This study had several limitations. First, the present study was retrospective as opposed to the well-designed, prospective, and randomized comparative clinical trials. Methodological bias could influence the reliability of these results. However, the use of propensity score matching might control for biases in our study. Second, it still lacks unified diagnostic criteria of CIE. We could not exclude the possibility of underdiagnosis of CIE in our cohort. Third, the low incidence of CIE and the small population of cases may limit the statistical power. Therefore, the effects of anesthetic drugs on CIE need further confirmation.

## Conclusion

The incidence of CIE was significantly higher in the general anesthesia group than in the local anesthesia group. Logistic regression analysis showed a positive association between general anesthesia and the risk of CIE, indicating that general anesthesia might be a risk factor for CIE.

## Data availability statement

The raw data supporting the conclusions of this article will be made available by the authors, without undue reservation.

## Ethics statement

The studies involving human participants were reviewed and approved by the Medical Ethics Committee of Ruijin Hospital Affiliated to Shanghai Jiao Tong University School of Medicine. Written informed consent to participate in this study was provided by the patient/participants or patient/participants' legal guardian/next of kin. Written informed consent was obtained from the individual(s) for the publication of any potentially identifiable images or data included in this article.

## Author contributions

ZZ and HN analyzed the data and were the major contributors in writing the manuscript. JZ and HJ provided assistance for the data acquisition and literature search. JH, DL, and LB carried out the manuscript preparation and editing. DL performed a manuscript review. All authors read and approved the final manuscript.
