# Supplementary material for: Association between general anesthesia and contrast-induced encephalopathy after endovascular treatment on neurovascular diseases
Source: Front Neurol. 2023 May 12;14:1146194. doi: 10.3389/fneur.2023.1146194 (PMC10218683; doi:10.3389/fneur.2023.1146194)
Supplement: Supplementary file 1 [file Table_1.DOCX]

Supplementary table 1 Dose of anesthetics used during anesthesia procedure and during intubation prolonged after endovascular treatment

| Anesthetics | Dose of induction | Dose during anesthesia | Dose during intubation prolonged after endovascular treatment |
| --- | --- | --- | --- |
| Propofol | 1.5-2.5 mg/kg | 5.0-7.5 mg/kg·h | / |
| Dexmedetomidine | 0.25-0.50 mg/kg | / | 0.2-0.3 ug/kg·h |
| Rocuronium | 0.6-1.2 mg/kg | 9-12 ug/kg·min or 0.1-0.2 mg/kg intermittently | / |
| Cisatracurium | 0.15-0.20 mg/kg | 1-2 ug/kg·min or 0.02 mg/kg intermittently | / |
| Fentanyl | 20-30 ug/kg | 20-30 ug/kg intermittently | / |
| Sufentanil | 0.25-0.50 ug/kg | / | / |
| Remifentanil | / | 0.025-0.100 ug/kg·min | / |
